# Lifting for Health

**Published:** 1875-12

**Authors:** 


### LIFTING FOR HEALTH.

A year or more ago we were visiting the house of a literary friend, a gentleman of prominence in the world of letters, and one capable of performing quite marvellous feats of composition ; doing, in fact, in a single day much more than most writers accomplish in a week of active work. Being a guest at his house for several days, we learned something of his method of labor, and discovered that he depended for that activity of brain and vigor of body which render rapid and long-continued literary work possible, upon a MANN'S REACTORY LIFTING MACHINE.

The gentleman to whom we allude, is Mr. Samuel Clemens, better known for his wonderfully humorous writings, as "Mark Twain." He kept his lifting machine convenient to his library, and after some hours of work at the desk he would set the sluggish life-current in motion by a few minutes' lifting. He assured us that the effect produced was that of strong exercise, without exhaustion ; a *condensation* of exercise such as could not be secured from gymnastics ; with a certain exhilaration of nerve and muscle and brain, obtainable from no other employment of physical effort. To the literary or other sedentary person, to whom time is always of great importance when the inspiration is on or there is important work in hand, a concentrated, handy system of exercise is is of infinite value. The danger is that at such periods exercise will be neglected altogether. Before the advent of the lifter, brain workers were in the constant practice of devoting themselves to protracted periods of work without exercise, to be followed, perhaps by excessive out-door activity. Dickens informed us that he would sometimes work ten hours a day for weeks at a time, when producing one of his stories, and would walk twenty or thirty miles a day when no labor pressed upon him. Doubtless this irregularity told upon his constitution, for nature abhors nothing so much as want of system. Lifting, as a nerve-stimulant and brain-tonic was not then in vogue; had it been, the world might not now mourn the untimely demise of one of its greatest geniuses.

Of the importance of some kind of physical exercise the intelligent world is fully aware. Yet perhaps no essential is so generally neglected. This neglect has been largely owing to the fact that to take sufficient exercise in the ordinary way– that is, of walking--involves a very serious loss of time. The need exists for exercise in a concentrated form ; exercise boiled down and bottled up for use at odd moments, and in brief intervals. This need is met by the properly constructed lifting device. The exercise which it affords is not of the hurried, exhausting type. It does not induce panting, and palpitation, and subsequent weariness. Yet it ultimately calls every muscle into action and imparts to it fulness, and symmetry, and power. Its greatest value rests in its graduated character, its cumulative quality. The feeblest lady, unable to lift a pitcher of water, may by a few moments daily use of this machine, acquire the power to lift hundreds of pounds. The increase is gradual, and is proportioned to the growing strength.

This lifting-exercise is a great beautifier in many ways. It gives roundness to the form, erectness to the carriage, and clearness to eye and complexion. It is an inveterate enemy to undue flesh, because it conduces to the healthy action of all the functions of life. It is a wonderful remedy for billiousness, indigestion and torpid stomachs and livers, because if persisted in, it refuses to allow the internal viscera to remain inactive and sluggish. It will cure more diseases than all the drugs under the sun. It never thwarts the plans of nature, but acts ever in harmony with them. For the gloomy and despondent and depressed, there is nothing so surel · and safely exhilarating.

The advantages to be gained from the judicious use of this system of cumulative exercise and cumulative power and cumulative health, may best be seen at the central establishment of the Health Lift Company, No. 46 East 14th Streel, Union Square, New York. To these pleasant rooms, hundreds of health-seekers of both sexes flock, and here, under the guidence of skilled advisers, they find health and strength and added grace. From this place, hundreds of these little machines are sent forth to exert their cheering, invigorating influence over the inmates of as many homes.

We have preached the Gospel of exercise, for many long years. We know that good exercise is as needful as good air or good food. Nothing can wholly take the place of sunlight and open-air life. The lifting machine of Mr. Mann, will not supply all the needs of humanity. As an adjunct to other right methods, it will do more, we are convinced,towards the building up of a strong and noble frame-work,a clear and vigorous intellect and a eheerful, generous habit of thought, than any other system of exercise now in general use. It has proved itself a wonderful renovator of diseased bodies as well as a marvellous brightener of languid, inert and over worked brains. Let our friends read the entertaining pamphlets of the Company which may be had for the asking, at its agencies in all large cities.

